# The China Initiative must end

**DOI:** 10.1126/sciadv.abo6563

**Published:** 2022-02-24

**Authors:** H. Holden Thorp

**Affiliations:** H. Holden Thorp is the Editor-in-Chief, *Science* Journals. Email: hthorp@aaas.org

**Figure:**
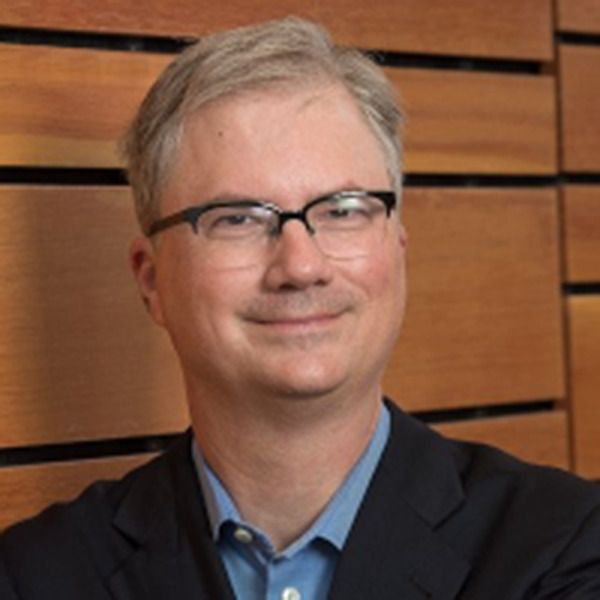
H. Holden Thorp

In the fall of 2018, declaring “enough is enough,” then-Attorney General Jeff Sessions launched the China Initiative which sought to address “new and evolving threats to our economy, not only defense and intelligence targets, but also universities and research institutions.” (*1*) The government felt that there was significant evidence of economic espionage by China against the US, and it must be addressed. Four years later, there is scant evidence that the amount of illegal activity turned up was worth the heartache and expense of running the initiative, and very little, if any evidence, was found showing that actual trade secrets were transferred prematurely to China. Enough really is enough—it is time to end this waste of resources and attention.

US federally funded research efforts—and those of many other countries—have always had an underlying nationalistic agenda—a point sometimes forgotten by the recipients of federal funding. But ever since Vannevar Bush argued for the creation of a federally funded scientific enterprise in 1945, there has always been a nationalistic motivation presented to Congress that success in science would lead to US economic, medical, and military success. Initially, this approach to the scientific enterprise was used to prevail over Germany and Japan. It was then used in relation to the former Soviet Union, and now China. Each year at the congressional budget hearings, representatives from federal science agencies and scientific societies present the case for more science funding which to many researchers would be considered shockingly nationalistic. In recent years, this involves talking about the rise of Chinese science and how it is a threat to US economic and military success.

What’s striking is the cognitive dissonance between this nationalistic message and the way that science is actually conducted. Scientific progress relies on collaboration, on recruiting the best possible talent to important scientific problems, and on publicizing these findings to the entire world. So setting up science as a competition with talented scientists in other countries and as harboring secrets that are not to be shared widely flies in the face of the core values of the scientific community. But year after year, scientific leaders go to Congress and request increases in funding using the rhetoric of nationalism, all while realizing that this approach is in conflict with the values and expectations of bench scientists. Further, when the yearly request for funding comes around, the agencies are always willing to adopt policies that put administrative burden and blame on individual institutions to better protect the overall enterprise. This doctrine of doing what it takes to get the money is nothing new; it has attended science policy for 75 years.

It is therefore no surprise that when the China Initiative was announced, the National Institutes of Health dutifully began sending letters to universities demanding they audit the China connections of prominent researchers. Most, but not all, of these researchers were Chinese or of Chinese descent, which has logically induced accusations that the China Initiative was racially motivated. Further, these efforts sought to determine whether researchers were receiving and disclosing funds from China even though there was no clear evidence that the failure to disclose such funding was an effective indicator of whether research knowhow was being inappropriately transferred. Instead, the whole effort became an exercise in determining whether correct forms were being filled out, something college professors are notoriously bad at doing, especially in recent years when the administrative burden of holding federal grants has escalated significantly.

The failure of the China Initiative to produce any results shows the serious flaws in the reasoning behind it. The recent acquittal of Gang Chen, who writes an editorial in *Science* this week about his experiences, shows how much damage has been done to the scientific enterprise with so little—in Chen’s case nothing—in return. So far, the initiative’s only significant conviction occurred when Harvard chemist Charles Lieber failed to report as income a bag of cash from his China activities and then didn’t explain it accurately to the FBI. As I have written elsewhere, Lieber certainly deserves to be convicted for these illegal acts, but even in this case, there was no evidence that scientific secrets were inappropriately transferred to China. Given Lieber’s robust record of scientific publication, it is hard to imagine how he would have been harboring any such secrets that he wasn’t planning to publicize widely.

There are actual incidents of scientific information being transferred to China prior to publication in the US. The most famous case involves the apparent theft of knowhow on metamaterials from David Smith’s laboratory at Duke University. It is entirely appropriate for the US intelligence apparatus to pursue cases such as this, but it is an immense overreaction to send university administrators into a frenzy auditing the forms of their productive faculty when they have so much work to do facilitating the research itself. More importantly, this effort has a chilling effect both on establishing legitimate and important collaborations with China, and even more importantly, it sends exclusionary and discriminatory signals to researchers of Chinese descent who are making such important contributions to research in the US.

What should be done? The federal government needs to wind down the China Initiative immediately and go back to managing the small number of legitimate issues on an episodic basis. Yesterday, the Department of Justice said they were retiring the name of the initiative but “broadening” the effort and not winding down any of the 2,000 cases that are currently open; that is unlikely to make much difference. The funding agencies need to stop the practice of piling more administrative burden on researchers and institutions every time Congress gets another erroneous idea about scientific research; this is where enough truly is enough. The institutions need to take a stronger stand against these efforts; MIT rightly stood by Gang Chen but this has not been the norm with most of these cases. And finally, researchers who accept federal grants need to understand that they are participating in an effort steeped in a nationalistic agenda over the last 75 years. Only by understanding the full context of how federal funding is achieved can faculty members participate in the conversation about how to improve both the quality of science and the humaneness and compassion of the system.
